# Probing Nanoscale Thermal Transport in Surfactant Solutions

**DOI:** 10.1038/srep16040

**Published:** 2015-11-04

**Authors:** Fangyu Cao, Ying Liu, Jiajun Xu, Yadong He, B. Hammouda, Rui Qiao, Bao Yang

**Affiliations:** 1 Department of Mechanical Engineering, University of Maryland, College Park, MD 20742, USA; 2 Department of Mechanical Engineering, Virginia Tech, Blacksburg, VA 24061, USA; 3 Center for Neutron Research, National Institute of Standards and Technology (NIST), 100 Bureau Drive, Stop 6102, Gaithersburg, MD 20899 USA

## Abstract

Surfactant solutions typically feature tunable nanoscale, internal structures. Although rarely utilized, they can be a powerful platform for probing thermal transport in nanoscale domains and across interfaces with nanometer-size radius. Here, we examine the structure and thermal transport in solution of AOT (Dioctyl sodium sulfosuccinate) in n-octane liquids using small-angle neutron scattering, thermal conductivity measurements, and molecular dynamics simulations. We report the first experimental observation of a minimum thermal conductivity occurring at the critical micelle concentration (CMC): the thermal conductivity of the surfactant solution decreases as AOT is added till the onset of micellization but increases as more AOT is added. The decrease of thermal conductivity with AOT loading in solutions in which AOT molecules are dispersed as monomers suggests that even the interfaces between individual oleophobic headgroup of AOT molecules and their surrounding non-polar octane molecules can hinder heat transfer. The increase of thermal conductivity with AOT loading after the onset of micellization indicates that the thermal transport in the core of AOT micelles and across the surfactant-oil interfaces, both of which span only a few nanometers, are efficient.

Nanoscale thermal transport, broadly defined as thermal transport in nanoscale domains and across nanoscale interfaces, plays a critical role in applications ranging from thermal management in microelectronics, cryopreservation, to thermal energy storage^1^,^2^,^3^,^4^. Because of the difficulties in device fabrication and materials characterization, experimental investigation of the thermal transport in nanoscale domains and across interfaces (especially if the interface itself has a radius of a few nanometers) is typically challenging. While this issue is partially alleviated by the tremendous advance in nanofabrication and ingenious design of thermal characterization schemes^5^,^6^,^7^,^8^,^9^, there remains a great need to develop effective methods for probing nanoscale thermal transport.

Surfactants are amphiphilic molecules featuring a head group and a tail group with opposite affinity to polar and apolar solvents, respectively. When surfactants are introduced in solvents at sufficiently low concentration, they exist in the form of well-dispersed monomers. As the surfactant concentration in the solvent exceed a certain threshold (often termed the critical micelle concentration, or CMC)^10^, surfactant molecules self-assemble into supramolecular structures with various shapes and sizes. One of the simplest supramolecular structure is the spherical micelle. For surfactant molecules self-assembled in oil, their micelles are also widely termed reverse micelles. Except when the solution is prepared in very dry environment, the micelles are usually swollen due to the adsorption of a trace amount of water from air into the hydrophilic (oleophobic) core of the micelle. A key property of the micelle is that their dimension can be tuned easily from a few to tens of nanometers by varying the choice of surfactant, the loading of the surfactant, and the temperature, *etc*.

Surfactant solutions, and in particular those containing micelles, have found wide industrial and research applications. For example, they have been used as model systems for studying dynamics of water in confinement, as working fluids for enhanced oil recovery, and as a tool for nanoparticle synthesis. However, the application of surfactant solutions in thermal transport has received only limited attention^11^, e.g., our recent work showed that swollen micellar solution can be a very effective heat transfer fluids for boiling^12^. Here we note that since the internal structure of surfactant solutions can be varied systematically from single monomers dispersed in a sea of solvents to self-assembled micelles with well-defined shape and nanoscale dimension, they can be used as a powerful platform for studying nanoscale thermal transport. In particular, it allows one to probe the thermal transport at the interface between a *single* head group of surfactant molecule and its surrounding fluid, within the nanoscale core of micelles, and at the interface between surfactant’s head/tail groups and the solvent, which may have a radius of just a few nanometers. The possibility to explore nanoscale thermal transport by studying the thermal transport in surfactant solutions has been recognized recently^13^,^14^. For example, using ultrafast transient absorption method, Schmidt *et al.* has shown that the transition of the meso-structure of surfactant molecules greatly alters interfacial thermal transport, i.e., the interfacial thermal conductance decreases as the surfactant concentration approaches the critical micelle concentration.

In the present work, we report a new study of nanoscale thermal transport using a surfactant solution as a platform. We investigate the evolution of the microstructure and the thermal conductivity of AOT/n-octane solutions as a function of the AOT loading. The thermal conductivity of the solutions with different AOT concentrations is measured using the 3 ω-wire method. The microstructure of the solution is determined using the small-angle neutron scattering (SANS) technique. A minimum thermal conductivity is observed at the CMC of the AOT/n-octane solution, which cannot be explained by the existing thermal conductivity theories for the mixtures, such as the effective medium theories. The thermal conductivity data are then interpreted with the help of molecular dynamics simulations to provide insight into thermal transport within nanoscale domains and at surfactant-oil interfaces with nanoscale radius. We note that the scheme used in the present study shares commonality with the ultrafast transient absorption method used by Schmidt *et al.*^13^. Both methods utilize thermal measurement of bulk materials exhibiting nanoscale structures. The 3 ω-wire method measures the thermal conductivity of the bulk fluid, and itself does not possess nanometer resolutions directly. So we used small-angle neutron scattering technique to determine the microstructure of the fluid, and used molecular dynamics simulation to explore thermal transport mechanisms across the micelles. Nevertheless, both our method and the ultrafast transient absorption method can provide insight into nanoscale thermal transport by interpreting bulk measurement data using models for thermal transport in heterogeneous materials. However, it might be difficult to apply the ultrafast transient absorption method to the surfactant solutions because there are no absorption “core’ or nanoparticles in them.

## Results

### Structural characterization of surfactant solutions

**Figure 1:**
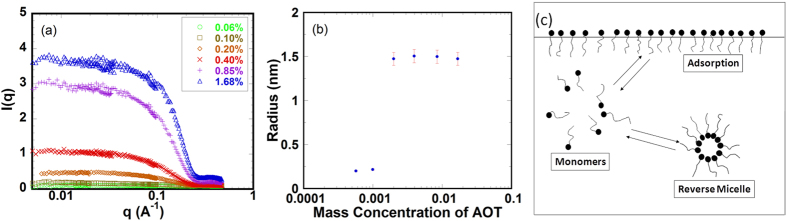
(**a**) SANS scattering intensity *I* versus scattering vector *q* for the AOT/octane solution. (**b**) The radius of the AOT micelles versus mass concentration of AOT in n-octane liquids. (**c**) A schematic showing the adsorption of AOT molecules at an oil/air interface, dissolution of AOT in oil in monomers, and self-assembly of AOT into micelles.

AOT was dissolved into n-octane liquids to form surfactant solutions. The microstructure of the solution was characterized using the SANS technique. Prior works showed that SANS can be used as a non-invasive, nanoscale probe of the structural transitions in the surfactant solutions^15^,^16^,^17^,^18^. Figure 1a shows the SANS data for the AOT/n-octane solutions, the scattering intensity *I* versus the scattering vector *q* = 4πsin(θ/2)/λ, where λ is the wavelength of the incident neutrons and θ is the scattering angle. The micelle size can be obtained by fitting these SANS data using the hard-sphere model in the IGOR PRO software under the protocol from NCNR NIST^16^,^19^,^20^,^21^. Figure 1b shows that reverse AOT micelles start to form at the AOT concentration ~0.1%; that is, the CMC for the AOT/n-octane system is ~0.1%. The reverse micelles have a radius of about 1.47 nm, indicating that each micelle consists of about 20 AOT molecules. At lower concentrations, AOT molecules exist in the form of monomers in non-polar octane solvent, but some molecules will be adsorbed at the air/solution interface and onto the walls of the containing vessel. When the AOT concentration exceeds CMC, AOT molecules aggregate to form micelles (see Fig. 1C).

### Thermal conductivity of surfactant solutions

**Figure 2:**
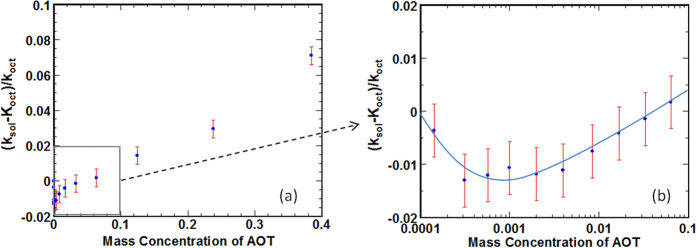
(**a**) Relative thermal conductivity of the AOT/octane solution versus AOT loading. (**b**) A zoom-in view on the thermal conductivity around the critical micelle concentration (CMC) of the AOT solution. The solid line is drawn as a guide for the eyes.

The raw experimental data for AOT/octane solutions with different AOT loadings are shown in Fig. 2a. The thermal conductivity of the pure octane is experimentally found to be 0.14 W/mK at room temperature, which is in good agreement with literature data^22^,^23^. Figure 2b shows the relative thermal conductivity in the AOT/octane solution as a function of the AOT loading. The relative thermal conductivity is defined as (*k*_*sol*_ − *k*_*oct*_ )*/k*_*oct*_, where *k*_*oct*_ and *k*_*sol*_ are the thermal conductivities of the neat n-octane and the AOT/octane solution fluids, respectively. Interestingly, a minimum thermal conductivity is seen around the critical micelle concentration (CMC) in the prepared AOT/octane solutions. The minimum thermal conductivity is also observed in other AOT solutions, such as AOT/poly-alpha-olefin solutions.

## Discussion

The non-monotonic dependence of the thermal conductivity of surfactant solutions on the AOT loading is not expected, at least from the perspective of classical effective medium theories such as the Maxwell-Garnett model^24^. Those theories have been developed for composites made of fillers dispersed in base materials, and they have been proven effective in numerous applications. They predict that, depending on the thermal conductivity, size, and shape of the filler as well as on the interfacial thermal conductance between the filler and the base material, as more fillers are introduced into a base material, the thermal conductivity of the composite either continuously increases or continuously decreases. However, we note that the application of the classical effective medium theories for surfactant solutions over the broad range of surfactant loading explored here is problematic for two reasons. First, the fillers (surfactant in this case) go through a structural transition as their loading is increased (from dispersed surfactant monomers to self-assembled micelles), which is not easily accounted for in the effective medium theories. Second, it is difficult to assign a value for the thermal conductivity of the fillers: although the thermal conductivity of AOT in solid form is well-known, the thermal conductivity of AOT monomers dispersed in oil is not well-defined and that for the AOT micelles is not known *a priori*.

**Figure 3:**
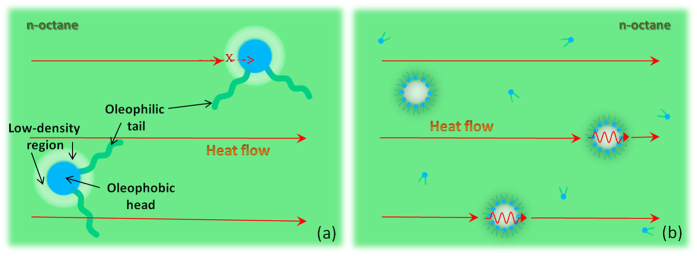
Schematics of the thermal transport in AOT solutions with AOT loading below (**a**) or above (b) CMC. (**a**) The interfacial region between AOT’s hydrophilic headgroup and the apolar octane molecules hinders the thermal transport near the headgroup, and thus reduces the thermal conductivity of the solution. (**b**) The hydrogen bonding network among the adsorbed water molecules and the AOT’s hydrophilic headgroups can provide a rapid thermal transport pathway and thus help increase the thermal conductivity of the micellar solution.

While we cannot use the classical effective medium theories to straightforwardly interpret the thermal conductivity data in Fig. 2, these data can provide valuable insight into the nanoscale thermal transport in liquid state. For AOT loading below the CMC, AOT molecules are dispersed in the octane fluids as monomers. Since prior experimental studies indicated that the thermal transport in a single AOT molecule is highly efficient^25^, the decrease of the thermal conductivity of the AOT solution as AOT loading increases suggests that the thermal transport between each AOT molecule and its surrounding octane fluid is not as effective as that between octane molecules, or equivalently, there exists a larger resistance for thermal transport between single AOT molecules and their surrounding octane molecules than that between octane molecules. While the mechanisms for the inefficient thermal transport between single AOT molecules and their surrounding octane molecules are not fully clear at present, such an effect is likely related to the non-ideal packing of octane molecules near the hydrophilic (i.e., oleophobic) headgroup of the AOT molecules (see Fig. 3). It is known that, near a hydrophobic group, the packing of water is less ideal than in the bulk due to the difficulty for water molecules to form hydrogen bonds in these conditions^26^,^27^. Along the same line, near the hydrophilic headgroup of an AOT molecule, the interactions between apolar octane molecules are not as strong as that of the intramolecular interactions between different sites on the head group and the intermolecular interactions between the Na^+^ counter-ion and the AOT’s headgroup. Therefore, the packing of octane molecules near an AOT’s headgroup will be less effective than in the bulk octane liquids, and this may lead to relatively ineffective thermal transport in the interfacial region near the AOT’s headgroup. As more AOT molecules are introduced into the octane liquids (but the CMC is not yet reached), the AOT headgroup-octane interfacial region expands and thus the thermal conductivity of the AOT solution decreases. In the above discussion, the contribution of the efficient thermal transport within each AOT molecule to the thermal transport is not taken into account, but the fact that the thermal conductivity of the AOT solution decreases as more AOT molecules are added suggests that the efficient intramolecular thermal transport in AOT plays a minor role in the overall thermal transport in the AOT solution. We hope that the new result shown here will prompt new, more thorough theoretical and experimental investigations that take into account both the intermolecular thermal transport (between AOT molecules and the interfacial octane; between the interfacial octane and the bulk octane) and the intramolecular thermal transport in AOT molecules and in octane molecules.

Once the AOT loading reaches CMC, AOT molecules self-assemble to form micelles with a radius of ~1.5 nm (see Fig. 1b). Above CMC, as more AOT molecules are introduced into the solution, the thermal conductivity of the AOT solution increases. Because micelles have well-defined geometry and are uniformly dispersed in octane liquids, the classical effective medium theory can be used to *qualitatively* understand the thermal transport in the micellar solution. Since the thermal conductivity of the micellar solution increases with AOT loading, using the Maxwell-Garnett model^24^, we postulate that the thermal transport within the AOT micelle is more efficient than that in bulk octane liquids and the thermal transport across the micelle-octane interfaces is efficient in general. To assess these postulations, we performed molecular dynamics (MD) simulations to study the thermal transport in AOT-octane solutions.

**Figure 4:**
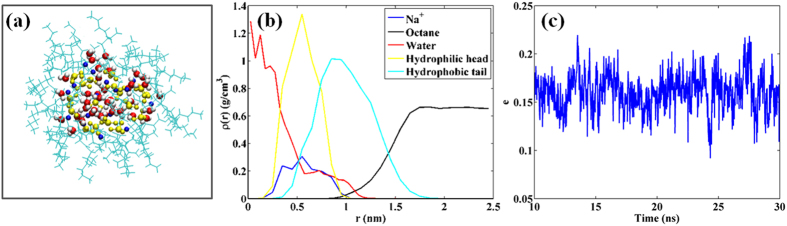
Structure of an AOT micelle in octane liquids. (**a**) A snapshot of a water-swollen micelle. The yellow balls denote AOT’s hydrophilic head and the cyan sticks denote AOT’s hydrophobic tail. The blue balls denote the Na^+^ ions. Red and white sticks denote the water molecule. The apolar octane liquids are not shown for clarity. (**b**) The radial density profile of the water molecules, the hydrophilic head and the hydrophobic tail of the AOT molecules. The center-of-mass of the micelle is taken as the origin. (**c**) The evolution of the eccentricity of the micelle during the last 20 ns of the simulation. The eccentricity *e* is defined as *e* = 1 − *I*_*min*_/*I*_*avg*_, where *I*_*min*_ and *I*_*avg*_ are the minimal and average moment of inertia of the micelle, resp*e*ctively^49^. *e* = 0 corresponds to a perfect sphere.

We begin by simulating the self-assembly of 17 AOT molecules in bulk octane liquids (see Methods). Since the AOT solution in our experiments was not prepared in a strictly dry environment, we expect some water molecules to be adsorbed in the spherical core of the micelle^28^. Therefore, 36 water molecules, corresponding to a low water-surfactant ratio of ~2:1, are also included in the system. Figure 4a shows a snapshot of the AOT micelle after a 30 ns of simulation. As expected, the hydrophilic sulfonate headgroups of the AOT molecules curl up to form a rather spherical core, their alkane chain face outwards and are in intimate contact with octane molecules. The hydrophilic core of the micelle is swollen by water molecules, which form hydrogen bonds with AOT’s sulfonate headgroups. The water molecules span a diameter of ~2 nm, while the head and tail groups can reach ~1 and ~1.5 nm from the center-of-mass of the micelle, respectively (see Fig. 4b). Therefore, the size of the micelle is similar to that in our experiments. The micelle remains nearly spherical once formed (see Fig. 4c). The fact that the micelle remains in a well-defined shape for tens of nanoseconds suggests that the self-assembly of AOT micelle is reproduced effectively by our MD simulations.

**Figure 5:**
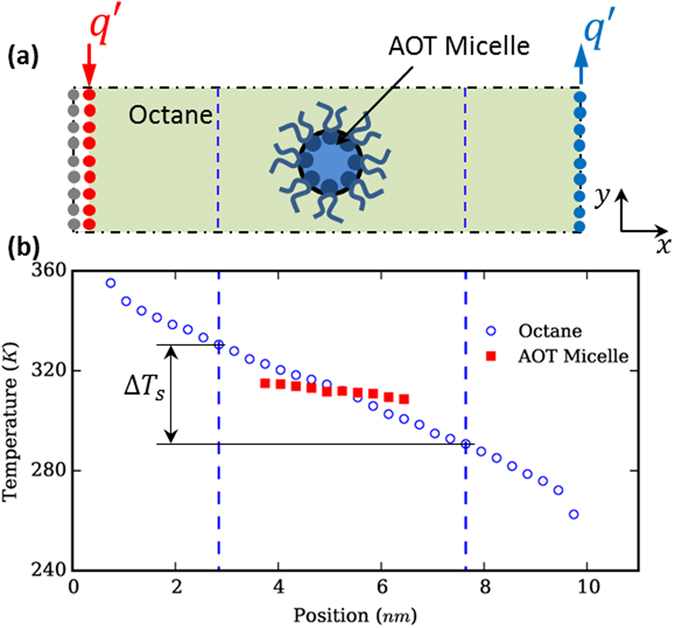
Molecular dynamics simulation of the thermal transport in micellar solutions. (**a**) A schematic of the MD system (not drawn to scale). The red and blue dots denote the two graphene layers used as heat source and sink, respectively. Since periodic boundary conditions are applied in all directions, a third graphene layer (shown as grey dots) is frozen at the edge of the simulation box to prevent thermal short circuiting. (**b**) The temperature profile of octane and AOT micelle in the simulation system. The nearly spherical micelle is located at the center of the cubic box (*L*_*x*_ = *L*_*y*_ = *L*_*z*_ = 4.8 nm) delineated by the two vertical dashed lines.

We next study the thermal transport through micelles using non-equilibrium MD simulations. A single pre-equilibrated micelle obtained above is placed inside an octane liquid bath as shown in Fig. 5a. The system is relaxed next for 500 ps in the NVT ensemble and then for another 500 ps in the NVE ensemble. After that heat is injected (removed) from the heat source (sink) at position far away from the micelle to generate a heat flux through the micellar solution (see Fig. 5a) and the system is evolved in the NVE ensemble. Figure 5b shows the temperature profiles of the AOT micelle and the octane liquids in the system after a steady state was reached. We observe that the temperature gradient within the micelle is much smaller compared to that in octane liquids, indicating that the thermal transport is more effective in the micelle than in octane liquids. Furthermore, the temperature profiles in Fig. 5b indicate that heat enters (leaves) the micelle from its left (right) side. This is consistent with the idea that is illustrated in Fig. 3b, i.e., the micelle serves as an efficient thermal transport pathway in the micellar solution. The effective thermal transport within the AOT micelle and across the micelle-octane interface is not very surprising. First, because of the strong interaction between AOT molecule’s sulfonate heads and the water molecules via hydrogen bonding and the ionic bonding between AOT’s head groups^29^,^30^,^31^, one could expect the thermal transport within the micelle to be very efficient. Second, prior studies of planar interfaces between hexane oil and the tail group of surfactant molecules have indicated that the thermal transport through such interfaces is extremely effective with an interfacial thermal conductance of ~400 MW/m^2^K^32^, hence one could expect the thermal transport across interfaces with similar chemical composition to be similarly efficient. However, both of these expectations are not without caveats: they are based on insights for thermal transport in bulk-like materials and across planar interfaces, while the thermal transport involving micelles occurs in molecularly-confined fluids and across interfaces with extremely large curvature. The present simulation, on the other hand, provides clear evidence that these expectations are qualitatively true.

Using the temperature profiles shown in Fig. 5b, we can also *approximately* calculate the effective thermal conductivity of micellar solutions. In our MD simulations, the cross-section of the simulation box is *L*_*y*_ × *L*_*z*_ = 4.8 nm × 4.8 nm. Therefore, we take the octane liquids and the AOT micelle within a cubic box (see Fig. 5b) that has a side width of *L*_x_ = 4.8 nm and encloses the micelle at its center to be a “virtual micellar solution”. Such a “virtual micellar solution”, with the micelle positioned at its center, can thus be considered as a representative portion of a bulk micellar solution. The thermal conductivity of the virtual micellar solution inside the cubic box is then computed using *k*_*s*_ = *q*′/(*L*_*y*_*L*_*z*_Δ*T*_*s*_), where *q*′ and Δ*T*_*s*_ are the heat flux and temperature drop across the cubic box, respectively. With the data shown in Fig. 5b and the above protocol, the surfactant loading and the average thermal conductivity inside the cubic box shown in Fig. 5, are found to be 16.1%wt and 0.119 W/mK, respectively. Since the thermal conductivity of the octane fluids modeled using the force fields adopted in our simulation is 0.111 W/m∙K (see Method), we estimate that the effective thermal conductivity of the micellar solution at a 16.1%wt AOT loading is enhanced by 6.3% compared to that of bulk octane liquids. Using the experimental data shown in Fig. 2, we obtain, through linear interpolation, that the enhancement of thermal conductivity by adding AOT to octane fluids is 2.0% at an AOT loading of 16.1%wt (linear interpolation is justified as the experimental thermal conductivity varies linearly with the AOT loading for loading from 8% to 24% (see Fig. 2)). Clearly, the enhancement of thermal conductivity predicted by the MD simulation is stronger than that inferred from our experimental measurements. The overestimation is likely due, in part, to the fact that the force fields used for water overestimates its thermal conductivity by about 30%^33^.

We note that it is desirable that MD simulations are performed at various AOT loadings so that effects of AOT loading on the thermal transport in surfactant solutions are understood more thoroughly. Such simulations, however, are very demanding for two reasons. First, for simulation with low AOT loadings, the simulation box will need to be large and hence the computational cost is high. For simulation with higher AOT loadings (≥25%), the experimental thermal conductivity data clearly deviates from the extrapolation from that measured at lower AOT loadings (see Fig. 2). A likely reason is that the surfactant solutions at such high AOT loading feature internal structure more complicated than spherical micelles. Reproducing such internal structure requires MD simulations to be performed for much longer than that is necessary for spherical micelles (tens of nanoseconds). Given these practical difficulties, we reserve these studies for future investigations.

**Figure 6:**
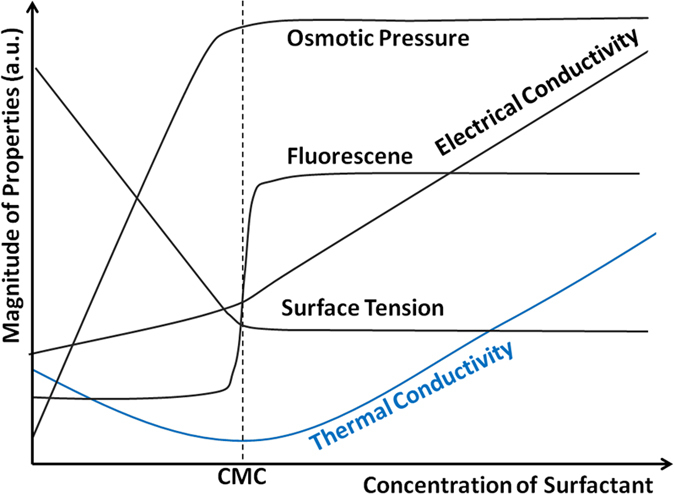
A schematic representation of the dependence of common physical properties of surfactant solutions on the surfactant concentration.

The observed thermal conductivity minimum at CMC in surfactant solutions not only provides a way to gain insight into nanoscale thermal transport through measurement of bulk properties, but also it can be used to determine the fundamental parameter CMC practically. As shown in Fig. 6, the dependence of many physicochemical properties of surfactant solutions on the surfactant concentration shows notable change near the CMC^34^,^35^,^36^,^37^,^38^,^39^,^40^. Therefore, by measuring the slope of the property-surfactant concentration curve, the CMC can be determined when a sudden change of slope is identified. In practice, such a method has some limitations if physical properties such as surface tension are used because the slope change is often continuous and it is not always easy to clearly identify a unique point of CMC. In comparison, the minimal thermal conductivity can be determined more readily and thus its measurement can be used to determine the CMC easily. This fact, together with the simplicity and rapidity of measuring the thermal conductivity of liquids, makes determining CMC via thermal conductivity measurement a useful alternative to the available methods.

## Conclusion

In this proof-of-concept study, we show that surfactant solutions can be a viable platform for probing nanoscale thermal transport. By systematically varying the loading of AOT surfactant in n-octane liquids, we found that the thermal conductivity of the solution shows a minimum at AOT loading corresponding to the CMC, at which surfactant molecules transition from dispersed monomers to self-assembled micelles inside the solution. The decrease of thermal conductivity with AOT loading in solutions below the CMC suggests that the thermal transport between individual oleophobic headgroup of AOT molecules and their surrounding non-polar octane molecules is not as effective as that in bulk octane liquids. The increase of thermal conductivity with AOT loading beyond the CMC indicates that the thermal transport within micelles with nanometer dimension and across the micelle-oil interfaces with extremely large curvature are both efficient, which is also confirmed in our MD simulations. Given that the nanoscale structure of surfactant solutions can be controlled by tuning many factors (e.g., the type of surfactants, temperature, *etc*.) and the thermal conductivity of bulk solutions is easily measured, surfactant solutions can be a powerful platform for probing nanoscale thermal transport that commonly requires advanced fabrication and characterization facilities. Finally, the fact that the minimal thermal conductivity of a surfactant solution occurs at the CMC suggests a new method for determining the CMC through thermal conductivity measurements.

## Methods

### Materials and sample preparation

Dioctyl sodium sulfosuccinate (98%) (AOT), n-octane (>99%), and deuterated n-octane (98 atom % D) were all purchased from Sigma-Aldrich. The desired amount of n-octane was placed in a glass tube, and then AOT was added to the glass tube. When the AOT loading exceeded the CMC, AOT/n-octane micelles were spontaneously formed by self-assembly of AOT molecules in the n-octane.

### SANS measurement

SANS measurements were carried out for the determination of the micelle size in the microemulsion. In this SANS experiment, samples were prepared using deuterated n-octane to achieve the needed contrast between the micelles and the solvent. SANS measurements are conducted on the NG-3 (30 m) beamline at the NIST Center for Neutron Research (NCNR) in Gaithersburg, MD. Samples were loaded into 2-mm quartz cells. The scattering intensity *I* was recorded as a function of the scattering vector *q *= 4π sin(θ/2)/λ, where λ is the wavelength of the incident neutrons and θ is the scattering angle. The approximation *q *= 2πθ/λ is used for SANS (due to the small angle θ). The error in droplet size is about 10%. The SANS data were processed using the IGOR software under the protocol from NCNR NIST.

### Thermal conductivity measurement

The 3ω-wire method was used to measure the thermal conductivity of liquids^41^,^42^. The 3ω-wire method is actually a combination of the 3ω-wire and the hot-wire methods. Similar to the hot-wire method, a metal wire suspended in a liquid acts both as a heater and a thermometer. However, the 3ω-wire method determines the fluid conductivity by detecting the dependence of temperature oscillation on frequency, instead of time. In the measurement, a sinusoidal current at frequency ω is passed through the metal wire and then a heat wave at frequency 2ω is generated in the liquid. The 2ω temperature rise of the wire can be deduced by the voltage component at frequency 3ω. The thermal conductivity of the liquid, *k*, is determined by the slope of the 2ω temperature rise of the metal wire^41^,^42^:


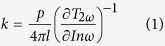


where *p* is the applied electric power, ω is the frequency of the applied electric current, *l* is the length of the metal wire, and *T*_2ω_ is the amplitude of temperature oscillation at frequency 2ω in the metal wire. The temperature oscillation *T*_2ω_ is given by


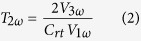


where V_1_ω is the amplitude of the voltage applied by the electrical power source across the heater, V_3_ω is the 3ω-voltage-component across the heater, and C_rt_ is the temperature coefficient of resistance of the heater. Calibration experiments of this thermal conductivity measurement method were performed in hydrocarbon (oil), fluorocarbon, and water at atmospheric pressure.

It is possible that the movement of charged particles or functional groups under an electric field would affect the measurement of the fluid thermal conductivity. However, we directly measured the electric current-voltage curves in pure oil, the AOT/oil solution, and air, and results indicate that the AOT/oil solution is as electrically insulating as the pure oil, and the AOT monomers and micelles are electrically neutral in the oil solution.

### MD simulations

To build an AOT micelle in n-octane liquids, 17 AOT molecules and 36 water molecules are packed into a spherical space of 4 nm in diameter using Packmol^43^. The resulting structure is next immersed in a box of n-octane measuring 5.1 × 5.1 × 5.1 nm^3^. The system is simulated using the LAMMPS code^44^ for 30 ns in the NVT ensemble (T = 300 K). The force fields for AOT are taken from those developed in Refs. 45, 46, 47. The octane molecules are modeled using the force fields in Ref. 48 and the water molecules are modeled using the SPC/E model. The equilibrated AOT micelle and its surrounding octane molecules are then placed inside a simulation box with a cross-section size of 4.8 nm × 4.8 nm in the *yz*-plane. The length of the simulation box is 10.76 nm in the *x*-direction and the center of the AOT micelle is located approximately at *x* = 5.29 nm. The system is next equilibrated in the NVT ensemble for 500 ps, and then in NVE ensemble for another 500 ps. To drive thermal transport through the system, two graphene layers positioned at *x* = 0.34 and 10.42 nm are used as heat source and sink, respectively. Since the system is periodic in all three directions, a third graphene layer is frozen at *x* = 0 to prevent thermal short circuiting. The MD system is evolved in the NVE ensemble for 7.5 ns, during which heat is added to (removed from) the carbon atoms in the heat source (sink) by velocity rescaling. The temperature profiles of AOT and octane fluids computed in the last 5 ns are shown in Fig. 5b.

## Additional Information

**How to cite this article**: Cao, F. *et al.* Probing Nanoscale Thermal Transport in Surfactant Solutions. *Sci. Rep.* **5**, 16040; doi: 10.1038/srep16040 (2015).

## References

[b1] Ordonez-Miranda J., Yang R. & Alvarado-Gil J. J. A crowding factor model for the thermal conductivity of particulate composites at non-dilute limit. J. Appl. Phys. 114, 064306 (7 Pages) (2013).

[b2] Yu W., Xie H., Wang X. & Wang X. Significant thermal conductivity enhancement for nanofluids containing graphene nanosheets. Phys. Lett. A 375, 1323–1328 (2011).

[b3] Zheng R. *et al.* Thermal percolation in stable graphite suspensions. Nano Lett. 12, 188–192 (2012). 22145977 10.1021/nl203276y

[b4] Ji Y., Wilson C., Chen H. & Ma H. Particle shape effect on heat transfer performance in an oscillating heat pipe. Nanoscale Res. Lett. 6, 296; 10.1186/1556-276x-6-296 (2011). 21711830 PMC3211362

[b5] Wang L. Q. & Wei X. H. Nanofluids: synthesis, heat conduction, and extension. J Heat Transfer, 131, 033102 (7 pages) (2009).

[b6] Cai W. *et al.* Thermal transport in suspended and supported monolayer graphene grown by chemical vapor deposition. Nano Lett. 10, 1645–1651 (2010). 20405895 10.1021/nl9041966

[b7] Yang J. *et al.* Enhanced and switchable nanoscale thermal conduction due to van der Waals interfaces. Nature Nanotech. 7, 91–95 (2012). 10.1038/nnano.2011.216 22157726

[b8] Cahill D. G. *et al.* Nanoscale thermal transport. II. 2003–2012. Appl. Phys. Rev. 1, 011305 (45 Pages) (2014).

[b9] Yang J. *et al.* Contact thermal resistance between individual multiwall carbon nanotubes. Appl. Phys. Lett. 96, 023109 (3 Pages) (2010).

[b10] Mitchell D. J. & Ninham B. W. Vesicles and microemulsions. J. Chemical Society, Faraday Transactions 2: Mol. and Chem. Phys. 77, 601–629 (1981).

[b11] Wang Y., Yu B., Zakin J. L. & Shi H. Review on drag reduction and its heat transfer by additives. Adv. in Mech. Eng., 3; 10.1155/2011/478749 (2011).

[b12] Xu J. J., Wu C. W. & Yang B. Thermal- and phase-change characteristics of self-assembled ethanol/polyalphaolefin nanoemulsion fluids. J. Thermophys. and Heat Transfer 24, 208–211 (2010).

[b13] Schmidt A. J. *et al.* Probing the gold nanorod-ligand-solvent interface by plasmonic absorption and thermal decay. J. Phys. Chem. C 112, 13320–13323 (2008).

[b14] Huang J., Park J., Wang W., Murphy C. J. & Cahill D. G. Ultrafast thermal analysis of surface functionalized gold nanorods in aqueous solution. ACS Nano 7, 589–597 (2013). 23230822 10.1021/nn304738u

[b15] Bergström M. & Pedersen J. S. Structure of pure SDS and DTAB micelles in brine determined by small-angle neutron scattering (SANS). Phys. Chem. Chem. Phys. 1, 4437–4446 (1999).

[b16] Hammouda B. SANS from polymers—review of the recent literature. J. Macromol. Sci., Part C: Polymer Rev. 50, 14–39 (2010).

[b17] Svensson B., Olsson U., Alexandridis P. & Mortensen K. A SANS investigation of reverse (water-in-oil) micelles of amphiphilic block copolymers. Macromolecules 32, 6725–6733 (1999).

[b18] Kumar P. & Mittal K. L. Handbook of microemulsion science and technology (Marcel Dekker, New York, 1999).

[b19] Xu J., Yang B. & Hammouda B. Thermal conductivity and viscosity of self-assembled alcohol/polyalphaolefin nanoemulsion fluids. Nano. Res.Lett. 6, 1–6 (2011). 10.1186/1556-276X-6-274 PMC3211338 21711807

[b20] Hammouda B. A new Guinier-Porod model. J. Appl. Crys. 43, 716–719 (2010).

[b21] Xu J., Yang B. & Hammouda B. Thermophysical properties and pool boiling characteristics of water in polyalphaolefin nanoemulsion fluids. *Proceedings of ASME Micro/Nanoscale Heat and Mass Transfer International Conference 2012,* *10.1115/MNHMT2012-75232* (2012).

[b22] http://www.engineeringtoolbox.com/octane-thermal-properties-d_1760.html. (Date of access:01/08/2015).

[b23] Li S., Maitland G. C. & Wakeham W. A. The Thermal conductivity of n-hexane and n-octane at pressures up to 0.64 gpa in the temperature range 34–90 °C. Berichte der Bunsengesellschaft für physikalische Chemie 88, 32–36 (1984).

[b24] Nan C. W., Birringer R., Clarke D. R. & Gleiter H. Effective thermal conductivity of particulate composites with interfacial thermal resistance. J. Appl. Phys. 81, 6692–6699 (1997).

[b25] Deak J. C., Pang Y. S., Sechler T. D., Wang Z. H. & Dlott D. D. Vibrational energy transfer across a reverse micelle surfactant layer. Science 306, 473–476 (2004). 15388896 10.1126/science.1102074

[b26] Chattopadhyay S. *et al.* How water meets a very hydrophobic surface. Phys. Rev. Lett. 105, 037803 (45 Pages) (2010). 20867810 10.1103/PhysRevLett.105.037803

[b27] Mezger M., Reichert H., Ocko B. M., Daillant J. & Dosch H. Comment on “How water meets a very hydrophobic surface”. Phys. Rev. Lett. 107, 249801 (1 Page) (2011). 22243027 10.1103/PhysRevLett.107.249801

[b28] Moulik S. P. & Rakshit A. K. Physicochemisty and applications of microemulsions. J. Surface Sci. and Technol. 22, 159–186 (2006).

[b29] Schoen P. A. E., Michel B., Curioni A. & Poulikakos D. Hydrogen-bond enhanced thermal energy transport at functionalized, hydrophobic and hydrophilic silica–water interfaces. Chem. Phys. Lett. 476, 271–276 (2009).

[b30] Hu M., Michel B. & Poulikakos D. Surface functionalization mechanisms of enhancing heat transfer at solid-liquid interfaces. J. Heat Transfer 133, 082401–082401 (2011).

[b31] Hu M., Goicochea J. V., Michel B. & Poulikakos D. Water nanoconfinement induced thermal enhancement at hydrophilic quartz interfaces. Nano Lett. 10, 279–285 (2009). 19958015 10.1021/nl9034658

[b32] Patel H. A., Garde S. & Keblinski P. Thermal resistance of nanoscopic liquid-liquid interfaces: Dependence on chemistry and molecular architecture. Nano Lett. 5, 2225–2231 (2005). 16277458 10.1021/nl051526q

[b33] Sirk T. W., Moore S. & Brown E. F. Characteristics of thermal conductivity in classical water models. J. Chem. Phys. 138, 064505 (11 Pages) (2013). 23425477 10.1063/1.4789961

[b34] Rosen M. J., Mathias J. H. & Davenport L. Aberrant aggregation behavior in cationic gemini surfactants investigated by surface tension, interfacial tension, and fluorescence methods. Langmuir 15, 7340–7346 (1999).

[b35] Binks B. P. Emulsion-type below and above the cmc in aot microemulsion systems. Coll. and Sur. A: Physicochem. Eng. Aspects. 71, 167–172 (1993).

[b36] Chattopadhyay A. & London E. Fluorimetric determination of critical micelle concentration avoiding interference from detergent charge. Analytical biochem. 139, 408–412 (1984). 10.1016/0003-2697(84)90026-5 6476378

[b37] Chatterjee A., Moulik S. P., Sanyal S. K., Mishra B. K. & Puri P. M. Thermodynamics of micelle formation of ionic surfactants: A critical assessment for sodium dodecyl sulfate, cetyl pyridinium chloride and dioctyl sulfosuccinate (Na salt) by microcalorimetric, conductometric, and tensiometric measurements. J. Phys. Chem. B 105, 12823–12831 (2001).

[b38] Lindman B. & Wennerström H. Micelles, in Micelles Vol. 87 Topics in Current Chemistry, 1–83 (Springer: Berlin Heidelberg,, 1980). 6987777

[b39] Mukerjee P. & Mysels K. J. Critical micelle concentrations of aqueous surfactant systems. (DTIC Document, 1971).

[b40] Turro N. J. & Yekta A. Luminescent probes for detergent solutions. A simple procedure for determination of the mean aggregation number of micelles. J. Am. Chem. Soc. 100, 5951–5952 (1978).

[b41] Xu J., Yang B. & Hammouda B. Thermophysical Properties and Pool Boiling Characteristics of Water in Polyalphaolefin Nanoemulsion Fluids. J. Heat Transfer 135, 091303 (6 pages) (2012).

[b42] Lee S. M., Matamis G., Cahill D. G. & Allen W. P. Thin-film materials and minimum thermal conductivity. Micro. Thermophys. Eng. 2, 31–36 (1998).

[b43] Martinez L., Andrade R., Birgin E. G. & Martinez J. M. PACKMOL: A package for building initial configurations for molecular dynamics simulations. J. Comput. Chem. 30, 2157–2164 (2009). 19229944 10.1002/jcc.21224

[b44] Plimpton S. Fast parallel algorithms for short-range molecular-dynamics. J. Comput. Phys. 117, 1–19 (1995).

[b45] Bjelkmar P., Larsson P., Cuendet M. A., Hess B. & Lindahl E. Implementation of the CHARMM force field in GROMACS: Analysis of protein stability effects from correction maps, virtual interaction sites, and water models. J. Chem. Theory Comput. 6, 459–466 (2010). 10.1021/ct900549r 26617301

[b46] Maitra A. Determination of size parameters of water aerosol ot oil reverse micelles from their nuclear magnetic-resonance data. J. Phys. Chem. 88, 5122–5125 (1984).

[b47] Abel S., Sterpone F., Bandyopadhyay S. & Marchi M. Molecular modeling and simulations of AOT-Water reverse micelles in isooctane: Structural and dynamic properties. J. Phys. Chem. B 108, 19458–19466 (2004).

[b48] Yi P. & Rutledge G. C. Molecular simulation of bundle-like crystal nucleation from n-eicosane melts. J. Chem. Phys. 135, 024903 (11 pages) (2011). 21766967 10.1063/1.3608056

[b49] Marchi M. & Abel S. Modeling the self-aggregation of small AOT reverse micelles from first-principles. J. Phys. Chem. Lett. 6, 170–174 (2015). 26263107 10.1021/jz5023619

